# Supplementary material for: Obesity Measured via Body Mass Index May Be Associated with Increased Incidence but Not Worse Outcomes of Immune-Mediated Diarrhea and Colitis
Source: Cancers (Basel). 2023 Apr 17;15(8):2329. doi: 10.3390/cancers15082329 (PMC10136922; doi:10.3390/cancers15082329)
Supplement: Supplementary file 1 [file cancers-15-02329-s001.zip › cancers-2249669-supplementary.pdf]

cancers MDPI

Table S1. Risk of developing of IMDC among different BMI subgroups.

	OR (CI)	*p*-value
BMI ≥ 30 vs. BMI ≤ 25	1.5(1.0-2.1)	0.029*
BMI between 25-30 vs. BMI ≤ 25	1.2(0.8-1.7)	0.426
BMI between 25-30 vs. BMI ≥ 30	0.8(0.6-1.1)	0.158

Abbreviations: IMDC – immune mediated diarrhea and colitis; BMI – body mass index.
